# Special Issue “Targeting of Functional Proteins in Disease Therapeutics: Enzyme Function and Inhibition Studies”

**DOI:** 10.3390/ijms27010070

**Published:** 2025-12-21

**Authors:** Sung-Kun Kim

**Affiliations:** Department of Natural Sciences, Northeastern State University, Tahlequah, OK 74464, USA; kim03@nsuok.edu

We are delighted to introduce the collection Targeting of Functional Proteins in Disease Therapeutics: Enzyme Function and Inhibition Studies, which showcases recent advances in understanding enzyme function and inhibition for disease treatment. Enzymes are often pivotal drug targets across oncology, metabolic disorders, and neurodegenerative conditions, with current strategies exploiting structure-based design, high-throughput experimental technologies, and computational modeling to improve the possibility of therapeutic innovation [1,2,3]. Additionally, enzymes are at the heart of cellular life, regulating processes ranging from metabolism to signaling, and orchestrating the complicated biochemical pathways that affect health and disease. Their roles and molecular specificity have made enzymes important targets for drug development, and recent progress in molecular biology, chemical synthesis, and computational science is radically transforming our ability to discover, validate, and optimize enzyme-targeted therapeutics [4].

Historically, targeting of enzymes has produced effective drugs for multiple diseases including infections, metabolic syndromes, and cancer. The rationale is that enzymes serve as biological switches, and their modulation can correct dysfunctional cellular states. Also, it should be mentioned that the landscape of targeting enzymes has evolved. The typical model of inhibitor design has given way to more sophisticated approaches, including allosteric modulation, covalent inhibition, and structure-based design that exploit the dynamic complexity of protein structures [5].

Recent surveys of FDA-approved therapeutics show that a large proportion of drugs—both small molecules and biologics—exert their action by targeting key enzymes or enzyme-linked receptors [6]. Advances in high-throughput screening (HTS) and omics technologies have dramatically increased the scale and pace at which enzyme-inhibiting compounds can be discovered [7,8]. The synergy between biochemical assays, chemical informatics, and even AI-driven modeling is currently empowering scientists to tackle challenges of selectivity and efficacy that have historically limited the clinical use of enzyme inhibitors.

The study by Gan et al. stands as a notable illustration of innovation in targeted enzyme therapeutics [1]. By leveraging CRISPR/Cas9 technology, the team established a luciferase reporter system in the HepG2 liver cell line to directly quantify the enzymatic activity of ASGR1, a receptor implicated in metabolism, liver disease, and cardiovascular health. High-throughput screening using this genetic model pinpointed promising inhibitors from microbial metabolite libraries, which were subsequently validated by molecular and biochemical methods. The approach reflects a broader trend wherein genetic engineering and omics are integrated with classical biochemistry to produce robust, physiologically relevant models for drug screening. Additionally, high-throughput platforms now enable the discovery not only of enzyme inhibitors but also of novel enzyme substrates, as seen in the exploration of glycosyltransferases and their role in detoxification and therapeutic modulation [3]. Mass spectrometric techniques have been adapted for rapid, accurate quantification of coenzymes such as NAD(H), offering powerful biomarkers for assessing disease states and treatment responses—especially in neurodegenerative and metabolic disorders [2].

Structure-based drug design stands at the most popular avenue for enzyme inhibitor development. The 3D structures made by cryo-electron microscopy, X-ray crystallography, and NMR analysis with computational tools such as molecular docking, molecular dynamics simulations, and even AI-powered binding prediction models allow unprecedented visualization and manipulation of enzyme active sites [9,10,11]. These methods help scientists to design inhibitors with improved selectivity and better pharmacokinetic profiles, minimizing toxicity and off-target effects. Moreover, databases of protein structures and known inhibitors greatly accelerate lead optimization, while cheminformatics show scientists to navigate huge chemical libraries. Machine learning models are increasingly used to predict bioactivity, anticipate drug resistance mutations, and suggest new chemical modifications, setting the stage for computational drug discovery that is validated rapidly in the lab and clinic [12].

Drug development for complex and chronic diseases increasingly seems to depend on enzyme-focused approaches. For example, in cardiovascular medicine, ASGR1 inhibition is emerging as a potent strategy for treating hypercholesterolemia and reducing atherosclerotic risk [1,13,14,15,16]. The demonstration that ASGR1 deficiency inhibits atherosclerosis in animal models emphasizes the clinical promise of targeting this receptor. Similarly, enzyme inhibitors can be foundational in oncology, where modulation of signaling kinases, proteases, and metabolic enzymes can halt tumor progression, induce apoptosis, or overcome resistance to established therapies [17].

The progress in targeting of enzymes is transforming therapeutic strategies in infectious disease, immunology, and metabolic disorders. More specifically, in infectious disease and immunology, enzymes are able to inhibit viral replication, alter immune signaling, and restore homeostasis in dysregulated inflammatory states [18]. Recent advances in understanding protease functions as antiviral defense and cell cycle regulation point to opportunities for diagnostic biomarker development and novel interventions in rare genetic disorders and cancers [19]. Also, as enzyme inhibitor candidates, natural products including those derived from plants, microbes, and marine organisms continue to influence enzyme inhibitor developments [20,21]. Due to their inherent chemical diversity and evolutionary refinement, natural products might translate to high activity and lower toxicity. This bioprospecting, coupled with HTS and cheminformatics, may unlock new sources of enzyme modulators for therapeutic applications in many human issues such as obesity, metabolic disease, and neurodegeneration.

Despite the recent progress, significant challenges remain. Obtaining high selectivity, minimizing side effects, overcoming metabolic instability, and circumventing acquired resistance are always ongoing concerns. Thus, the future of enzyme targeting in therapeutics will likely be shaped by advances in (1) multi-target inhibitors designed to simultaneously affect more than one pathway to attack disease complexity and heterogeneity; (2) AI-driven drug discovery that automates chemical design, virtual screening, and molecule optimization for rapid preclinical progression; (3) chemical biology approaches that develop small-molecule probes; (4) personalized medicine strategies that tailor enzyme-inhibiting therapies to individual genetic and metabolic profiles for optimizing efficacy and safety; and (5) synthetic biology and bioengineering innovations that create artificial enzymes with novel functions to utilize both therapy and diagnostics.

In conclusion, the growth of enzyme-focused drug discovery reflects collaboration among academia, industry, and regulatory agencies. Large-scale consortia accelerate the sharing of data, best practices, and technical innovations. Training in molecular biology, computational science, and pharmacology is increasingly interdisciplinary, preparing the next generation of scientists to address therapeutic challenges. The convergence of molecular biology, synthetic chemistry, computational approaches, and clinical medicine is driving rapid progress in enzyme-targeted therapeutics. Finally, with ongoing investment, transparent data sharing, and adaptive clinical trial design, we aim to develop promising innovative therapies, achieve improved patient outcomes, and gain an expanded understanding of the biological systems that drive human health and disease.
